# Compound Heterozygous *KCNQ1* Mutations Causing Recessive Romano–Ward Syndrome: Functional Characterization by Mutant Co-expression

**DOI:** 10.3389/fcvm.2021.625449

**Published:** 2021-02-22

**Authors:** Antonia González-Garrido, Mayra Domínguez-Pérez, Leonor Jacobo-Albavera, Omar López-Ramírez, José Guadalupe Guevara-Chávez, Oscar Zepeda-García, Pedro Iturralde, Alessandra Carnevale, Teresa Villarreal-Molina

**Affiliations:** ^1^Laboratorio de Genómica de Enfermedades Cardiovasculares, Instituto Nacional de Medicina Genómica, Mexico City, Mexico; ^2^Cátedras CONACyT, Consejo Nacional de Ciencia y Tecnología, Mexico City, Mexico; ^3^Department of Neurobiology, University of Chicago, Chicago, IL, United States; ^4^Departamento de Electrofisiología, Instituto Nacional de Cardiología “Ignacio Chávez”, Mexico, Mexico; ^5^Laboratorio de Enfermedades Mendelianas, Instituto Nacional de Medicina Genómica, Mexico, Mexico

**Keywords:** *KCNQ1*, long-QT syndrome, IKs, electrophysiology, A300T, P535T, recessive Romano-Ward syndrome

## Abstract

Next Generation Sequencing has identified many *KCNQ1* genetic variants associated with type 1 long QT or Romano-Ward syndrome, most frequently inherited in an autosomal dominant fashion, although recessive forms have been reported. Particularly in the case of missense variants, functional studies of mutants are of aid to establish variant pathogenicity and to understand the mechanistic basis of disease. Two compound heterozygous *KCNQ1* mutations (p.A300T and p.P535T) were previously found in a child who suffered sudden death. To provide further insight into the clinical significance and basis for pathogenicity of these variants, different combinations of wildtype, A300T and P535T alleles were co-expressed with the accessory β-subunit minK in HEK293 cells, to analyze colocalization with the plasma membrane and some biophysical phenotypes of homo and heterotetrameric channels using the patch-clamp technique. A300T homotetrameric channels showed left-shifted activation V_1/2_ as previously observed in *Xenopus* oocytes, decreased maximum conductance density, slow rise-time_300ms_, and a characteristic use-dependent response. A300T slow rise-time_300ms_ and use-dependent response behaved as dominant biophysical traits for all allele combinations. The P535T variant significantly decreased maximum conductance density and Kv7.1-minK-plasma membrane colocalization. P535T/A300T heterotetrameric channels showed decreased colocalization with plasma membrane, slow rise-time_300ms_ and the A300T characteristic use-dependent response. While A300T left shifted activation voltage dependence behaved as a recessive trait when co-expressed with WT alleles, it was dominant when co-expressed with P535T alleles.

**Conclusions:** The combination of P535T/A300T channel biophysical properties is compatible with recessive Romano Ward syndrome. Further analysis of other biophysical traits may identify other mechanisms involved in the pathophysiology of this disease.

## Introduction

In mammalian hearts, the slow delayed potassium rectifier current (IKs) largely contributes to shape the repolarization phase of the ventricular action potential. IKs results from the co-assembly of the Kv7.1 channel complex, consisting of four pore-forming α subunits encoded by the *KCNQ1* gene and accessory β minK subunits encoded by *KCNE1*, with variable stoichiometry (1:4–4:4) (1–5). Heterozygous *KCNQ1* mutations resulting in decreased or total loss of function of the Kv7.1 channel cause autosomal dominant Romano-Ward or type 1 long QT syndrome (LQTS). These patients are susceptible to malignant cardiac arrhythmia, which may cause syncope, seizures, and sudden death, frequently in young and/or apparently healthy individuals (6). Homozygous or compound heterozygous *KCNQ1* mutations cause Jervell Lange-Nielsen syndrome, a recessive form of LQTS with severe QT prolongation and congenital sensorineural deafness (7–11). Patients with mutations on both *KCNQ1* alleles, a prolonged QT interval and normal hearing suffer from recessive Romano-Ward syndrome and are considered a high-risk subgroup (12).

Next Generation Sequencing has identified many *KCNQ1* genetic variants associated with type 1 LQTS. However, particularly in the case of missense variants, establishing pathogenicity remains challenging. The American College of Medical Genetics and Genomics and the Association for Molecular Pathology (ACMG/AMP) published and recently updated guidelines to standardize the interpretation of genetic variants (13, 14), which include functional characterization as a criterion to help define pathogenicity. The standard method to study functional consequences of ion channel genetic variants is the electrophysiological characterization of mutant channels in heterologous expression systems using patch clamp.

We recently reported the case of a child with normal hearing who suffered sudden death, found to be compound heterozygous for *KCNQ1* mutations (P535T/A300T), suggesting recessive Romano-Ward syndrome (15). Some of the electrophysiological properties of the A300T Kv7.1 channel were previously studied in *Xenopus* oocytes, and this mutation was considered as pathogenic only in the homozygous state (16). The P535T mutation was initially classified as of unknown clinical significance (VUS) according to the ACMG/AMP criteria (14). Protein modeling had predicted that the P535T mutation would disrupt the formation of a calmodulin-binding site by steric hindrance, which might prevent trafficking to the plasma membrane (15). To provide further insight into the clinical significance and the mechanistic basis for pathogenicity of these mutations, we studied some biophysical phenotypes of Kv7.1, A300T, and P535T homotetrameric and heterotetrameric channels, and the colocalization of these channels with minK and the plasma membrane. The P535T mutation was found to decrease Kv7.1-minK-plasma membrane colocalization and maximum conductance density. In addition, we further explored possible electrophysiological mechanisms by which the A300T mutation may contribute to the LQTS phenotype, and characterized biophysical properties of P535T/A300T heterotetrameric channels, leading us to conclude that these mutations are compatible with recessive Romano-Ward syndrome in the compound heterozygous child.

## Materials and Methods

The family of the compound heterozygous *KCNQ1* P535T/A300T child who suffered sudden death was previously described (15). The mother was an asymptomatic P535T heterozygous carrier, with a QTc that was borderline at rest, but prolonged during exercise. The father and only sibling were A300T heterozygous carriers, asymptomatic, and had normal QTc intervals on the ECG. The biophysical properties of IKs currents produced by different Kv7.1 channels were analyzed by co-expressing WT, A300T, and P535T homo and heterotetrameric channels in HEK293 cells.

### Site-Directed Mutagenesis

*KCNQ1* (NM_000218) tagged with GFP and *KCNE1* (NM_001127670) tagged with RFP plasmids were acquired from Origene (Rockville, MD, USA). The A300T and P535T mutations were cloned in the *KCNQ1* plasmid using QuickChange II XL Site-Directed Mutagenesis Kit (Agilent Technologies; Santa Clara, CA, USA) following manufacturer's instructions. Mutant primers were designed using QuikChange Primer Design tool: A300T-FW: 5′-ccaccacagcgcatccgtgtagctgccgaactc-3′; A300T-RV: 5′-gagttcggcagctacacggatgcgctgtggtgg-3′; P535T-FW: 5′-ccgcacatcgtaagtcttccgcgcttgct-3′, and P535T-RV: 5′-agcaagcgcggaagacttacgatgtgcgg-3′. The presence of the mutations was confirmed by Sanger sequencing.

### Cell Culture and Transfection

Human embryonic kidney cells (HEK293) were kindly provided by Dr. Ricardo Félix Grijalva. Cells were maintained in Dulbecco's modified Eagle's medium (DMEM) supplemented with 10% FBS (Hyclone Laboratories Inc; Logan, UT, USA), 100 U/ml penicillin and 100 μg/ml streptomycin (Gibco; Waltham, MA, USA), in a humidified 5% CO_2_ atmosphere at 37°C. Cells at 60–80% of confluence were used to transiently transfect both channel complex subunits (*KCNQ1* and *KCNE1*) in a 1:1 ratio, using 1.5 μg of each construct. Transfection was made with Lipofectamine™ LTX Reagent with PLUS™ Reagent (Invitrogen; Carlsbad, CA, USA) according to manufacturer's instructions. Twenty-four hours after transfection, cells were seeded on poly-D-lysine-coated glass coverslips at 2 × 10^4^ cells. Electrophysiological recordings were performed 2 h after seeded to ensure adhesion to coverslip.

### Electrophysiological Recordings

Whole-cell recordings were made at room temperature using borosilicate pipettes (WPI; Worcester, MA, USA) with 3–5 MΩ resistance in standard solutions. All reagents were purchased from Sigma Aldrich (St. Louis, MO, USA) unless otherwise indicated. In all experiments, the external solution was (in mM) 145 NaCl, 5 KCl, 1.3 CaCl_2_, 1 MgCl_2_, 0.7 NaH_2_PO_4_, and 10 HEPES; plus ~6.5 mM NaOH to bring pH to 7.4 and osmolality to ~295 mmol/kg. The pipette (internal) solution was a conventional KCl solution: (in mM) 135 KCl, 7 NaCl, 0.1 CaCl_2_, 2 MgCl_2_, 3 Na_2_ATP, 10 EGTA, and 10 HEPES; plus ~33 mM KOH to bring pH to 7.3 and osmolality to ~300 mmol/kg. The patch clamp amplifier Multiclamp-700B and D-A/A-D converter Digidata 1550A (Molecular Devices; San Jose, CA, USA) were controlled by pClamp 10.5 (Molecular Devices). Capacitive currents were electronically nulled. Series resistances ranged from 3 to 15 MΩ (mean 6 ± 0.4 MΩ, *n* = 61) and were compensated 76 ± 0.4% for a mean residual value of ~1.45 MΩ. Potentials were corrected for a liquid junction potential of −0.5 mV, calculated with JPCalc software (17) as implemented by Clampex 10.5 (Molecular Devices). Cells were held at −80.5 mV.

#### Whole-Cell Current Analyses

Voltage dependence of whole-cell currents was quantified by constructing activation (conductance density-voltage) curves from data collected using a voltage protocol consisting of an iterated series of 5.5 s test steps from a holding potential of −80.5 to 99.5 mV, that activates the slow outwardly rectifying K current (IKs). Steady-state conductances of IKs were calculated from tail currents at −40.5 mV, divided by driving force (the difference between V step and the current's reversal potential), mean across all cells, plotted against the test step voltage, and fitted with a Boltzmann function (Equation 1).

$$\begin{array}{l} G\left(V\right)=\frac{G_{\min}-G_{\max}}{1+e^{\left(V-V_{1/2}\right)/S}}+G_{\max} \end{array}$$

where G(V) is conductance at voltage V, Gmin, and Gmax are minimum and maximum conductances, V_1/2_ is the voltage corresponding to half-maximal activation, and S is the voltage corresponding to an e-fold increase in G(V). Curve-fitting and statistical analyses were performed with OriginPro software (OriginLab; Northampton, MA, USA). Parameters of curve fits (V_1/2_, Gmax/Cm, S) were compared for all experimental series, number of cells per group varied from 6 to 14. Gmax/Cm represents the maximum conductance density.

#### Rise Time

Rise time refers to the time required for a signal to change from a given low value to a given high value. Here, these values were 10 and 90% of the step height, as typically applied. To compare current activation time courses (kinetics) of different homo and heterotetrameric channels, rise time of currents activated from 0 to 100 mV steps was measured during the first 300 ms (rise-time_300ms_), within the physiological human ventricular action potential duration range.

#### Use-Dependent Response

To assess the response of all multimeric channels during a stimulation at a normal heart rate, a 300 ms step at 49.5 mV from a holding potential of −80.5 mV was delivered 70 times during 1 min, 1.17 Hz. Plots of the end-step normalized current as a function of the step number were built to measure the rise-time of the response.

### Confocal Microscopy and Image Analysis

To investigate the colocalization of Kv7.1 WT, A300T, and P535T homo and heterotetrameric channels with minK and the plasma membrane, HEK293 cells transiently transfected with *KCNE1* and different combinations of WT, A300T, and P535T *KCNQ1* plasmids were seeded on poly-D-lysine-treated coverslips at 2 × 10^4^ cells per coverslip. Twenty-four hours after transfection, cells were washed with 1X PBS and fixed in 4% PFA. Fixed cells were treated with CellMaskTM Plasma Membrane Stain-Deep Red fluorophore (ThermoFisher Scientific; Waltham, MA, USA) following the manufacturer's protocol, and were observed with an LSM 780 NLO confocal microscope (Carl Zeiss; Jena, Germany). All images are representative of at least three independent experiments.

As previously mentioned, *KCNQ1* and *KCNE1* plasmids were tagged with GFP and RFP, respectively. Only cells expressing both GFP and RFP were selected for image analysis. Colocalization of the three signals (GFP, RFP, and Deep-Red) was quantified using the threshold overlap score (TOS), where TOS = 1 indicates colocalization, TOS = 0 indicates non-colocalization and TOS = −1 indicates anti-colocalization (18). Images were analyzed with Fiji software (https://imagej.net/Fiji) and EZColocalization plugin (19).

### Statistical Analysis

Data are expressed as mean ± SEM. The significance of differences between means was assessed with one-way ANOVA for individual parameters and Two-way ANOVA for curves, both followed by *post-hoc* Tukey's tests of significance.

## Results

### Activation Parameters

**Figure 1:**
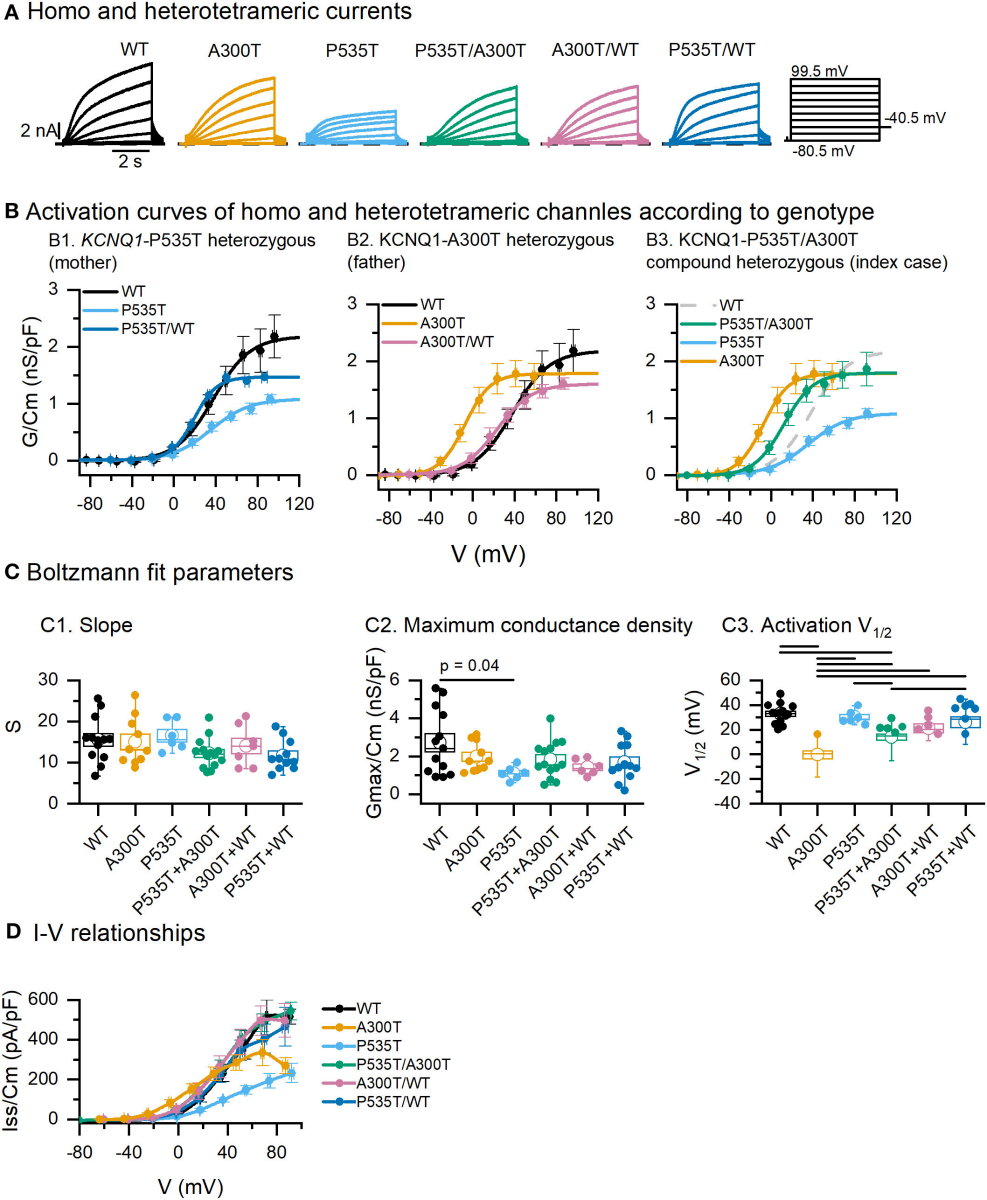
Activation parameters of homo and heterotetrameric channels. **(A)** Exemplar current traces resulting from co-expression of minK and WT, A300T and/or P535T Kv7.1 α subunits. **(B)** Activation curves obtained from tail currents. Circles represent mean ± SEM values and lines represent Boltzmann equation fits. **(C)** Boltzmann fit parameters for tetrameric channels. Empty circles represent means and boxes represent SEM values; full circles represent raw data. **(D)** Averaged steady-state I–V relationships of tetrameric channels. All differences marked in C1 were significant, *p* values for significant differences in C1 and D are shown in Table 1. Conductance-voltage (G-V) curves fitted to averaged data from WT (*n* = 13), A300T (*n* = 10), P535T (*n* = 6), P535T/A300T (*n* = 14), A300T/WT (*n* = 7), and P535T/WT (*n* = 11) cells.

Activation curves and kinetics are presented assuming the possible combinations of Kv7.1 homo and heterotetrameric channels (Figure 1A) expressed for three different genotypes (P535T/WT representing the mother, A300T/WT representing the father, and P535T/A300T representing the index case).

#### P535T/WT Kv7.1-minK Channel Complexes (Mother)

Activation curves for WT, P535T, and P535T/WT channels were similar in terms of their voltage dependence (mean V_1/2_ and S values were not significantly different, Figures 1B1,C1). Nonetheless, mean maximum conductance density (Gmax/Cm) of the P535T channels (1.11 ± 0.15 nS/pF, *n* = 6) was lower than WT channels (2.72 ± 0.48 nS/pF, *n* = 13; *p* = 0.04), but not significantly different from P535T/WT channels (1.68 ± 0.29 nS/pF; *p* = 0.9. Figures 1B1,C2; Table 1). No significant differences in S were found among mutant and WT proteins (Figure 1C3).

**Table 1:** *P* values for activation V_1/2_ and Iss/Cm comparisons among different channels.

**${\text{V}}_{\frac{1}{2}}$ comparisons**	***p***	**Iss/Cm comparisons**	***p***
A300T vs. WT	3.07E-8	A300T vs. WT	0.008
A300T vs. A300T/WT	7.64E-4	A300T vs. P535T/A300T	3.73E-4
A300T vs. P535T	3.07E-6	P535T vs. WT	5.91E-8
A300T vs. P535T/WT	1.92E-6	P535T vs. P535T/A300T	2.07E-10
A300T vs. P535T/A300T	0.01	P535T vs. A300T/WT	2.63E-5
P535T/A300T vs. WT	9.93E-5	P535T vs. P535T/WT	4.29E-4
P535T/A300T vs. P535T/WT	0.04		
P535T/A300T vs. P535T	0.02		

*Only statistically significant comparisons are shown*.

#### A300T/WT Kv7.2-minK Channel Complexes (Father)

Voltage dependence of the A300T homotetrameric channel was significantly left-shifted (V_1/2_: −0.21 ± 3.26 mV, *n* = 10) compared with WT homotetrameric (V_1/2_ = 32.96 ± 2.26 mV, *n* = 13; *p* = 3.07E-8) and A300T/WT heterotetrameric channels (V_1/2_: 21.17 ± 3.46 mV, *p* = 7.64E-4) (Figures 1B2,C1; Table 1). No significant differences in the voltage dependence of WT and A300T/WT channels were observed. No significant differences in S or Gmax/Cm were found among mutant and WT proteins (Figures 1C2,3).

#### P535T/A300T Kv7.1-minK Channel Complexes (Index Case)

The activation of P535T/A300T channels was significantly left-shifted (V_1/2_: 13.96 ± 2.31 mV, *n* = 14) compared with WT (V_1/2_ 32.96 ± 2.26 mV, *n* = 13; *p* = 9.93E-5) and P535T channels (V_1/2_: 30.22 ± 2.27, *n* = 6; *p* = 0.017); but significantly more positive than A300T channels (V_1/2_: −0.21 ± 3.26 mV, *n* = 10; *p* = 0.013) (Figures 1B3,C1; Table 1).

### Activation Kinetics

**Figure 2:**
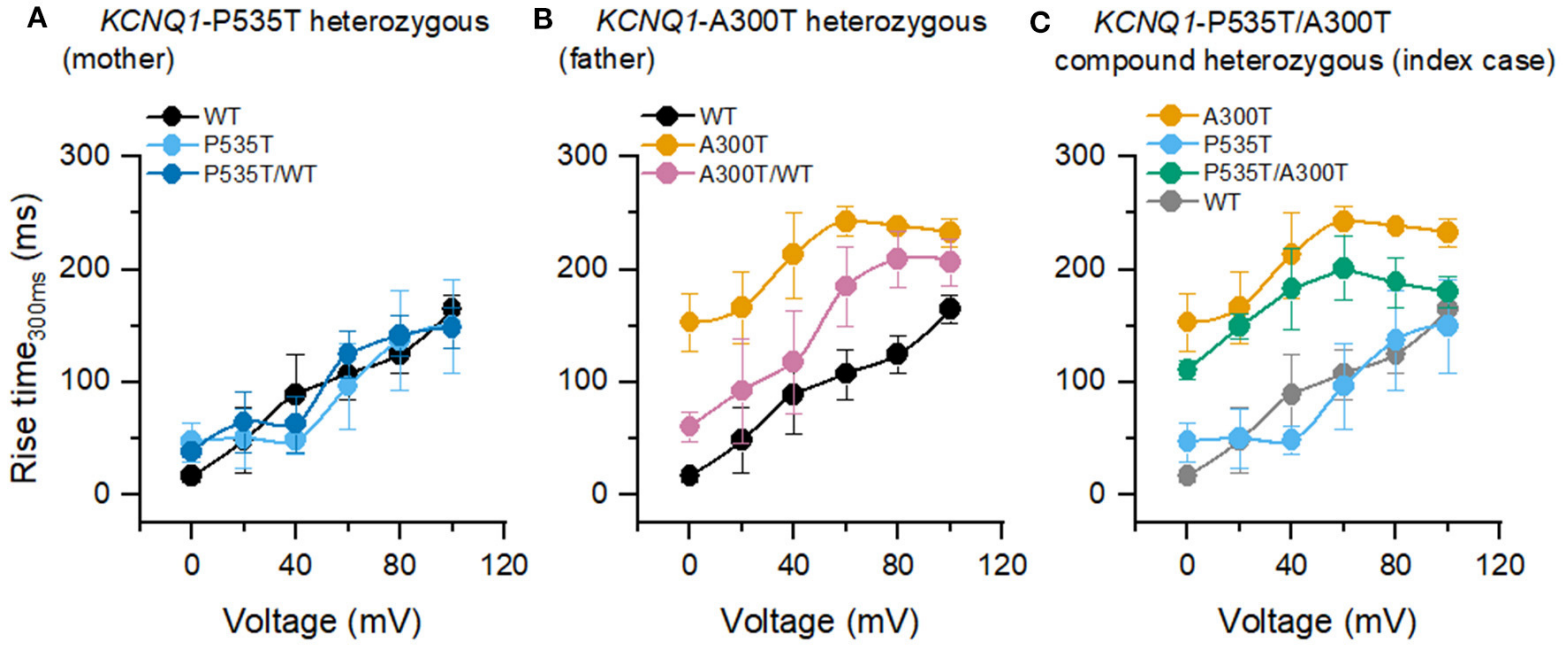
Rise time_300ms_ of homo and heterotetrameric channels. **(A)** P535T (*n* = 6) and P535T/WT (*n* = 7) currents shared similar rise-time_300ms_ values with WT (*n* = 13) currents. **(B)** A300T (*n* = 5) and A300T/WT (*n* = 8) currents were significantly slower than WT currents. **(C)** A300T and P535T/A300T (*n* = 8) currents were significantly slower than P535T and WT currents. WT rise-time_300ms_ values are shown in gray for comparison only.

Rise-time_300ms_ of currents from A300T-containing channels were significantly slower than those from WT, P535T, and P535T/WT channels (Figure 2; Table 2). On the other hand, rise-time_300ms_ of currents from P535T and P535T/WT channels were similar to those from WT channels.

**Table 2:** *P* values for rise-time300_ms_ comparisons among different channels.

**Rise-time_**300ms**_ comparisons**	**p**
WT vs. A300T	2.4E-7
WT vs. A300T/WT	0.0086
WT vs. P535T/A300T	0.0001
A300T vs. P535T	6.3E-7
P535T vs. P535T/A300T	0.0001

*Only statistically significant comparisons are shown*.

### WT and Mutant IKs Responses to Normal Heart Rate-Like Stimulation

**Figure 3:**
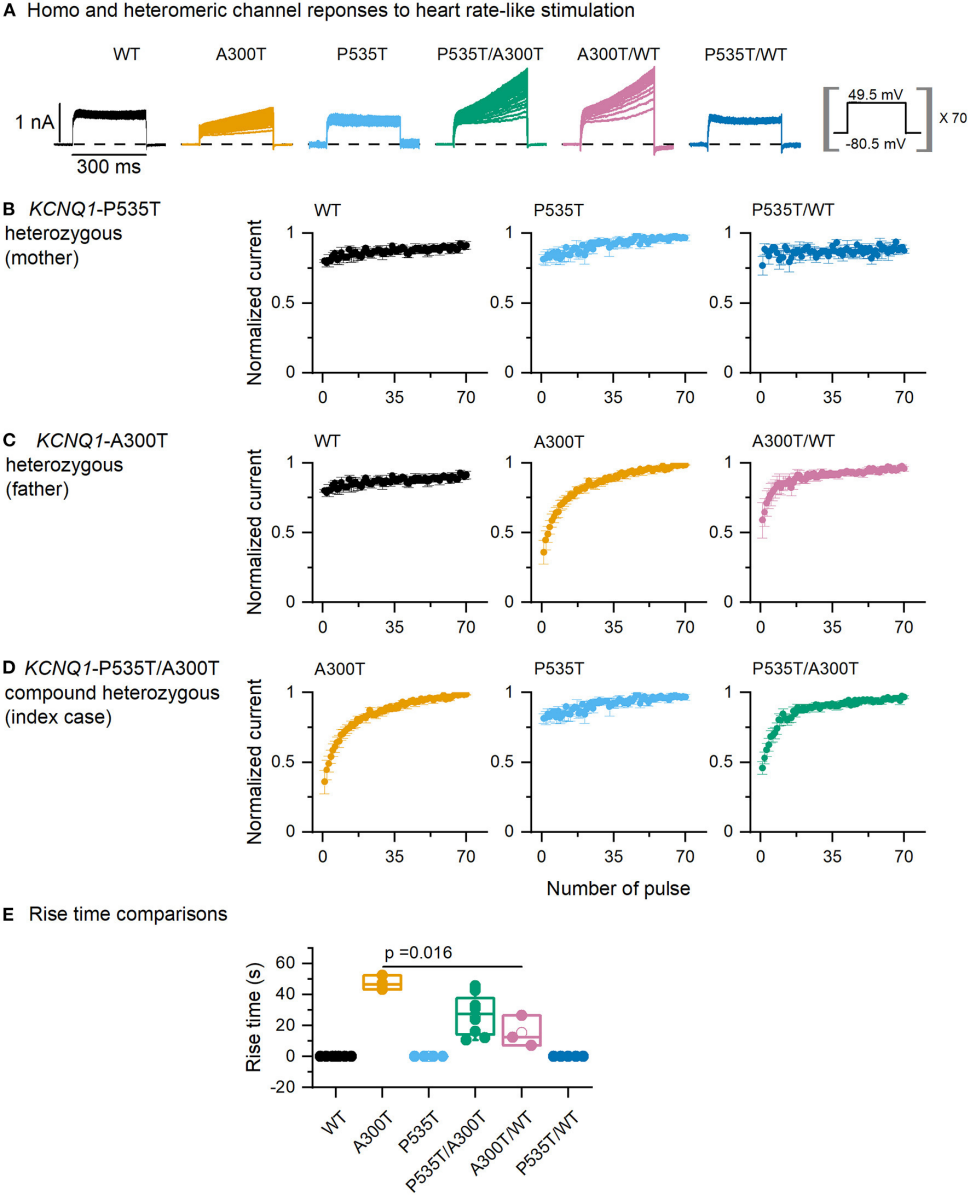
Current responses to normal heart rate-like stimulation. **(A)** Currents from homo and heterotetrameric channels. **(B–D)** End-step normalized current as a function of step number for P535T heterozygous (WT, P535T, and P535T/WT channels, all curves were similar), A300T heterozygous (WT and A300T and A300T/WT channels) and P535T/A300T compound heterozygous (P535T, A300T, and P535T/WT channels) genotypes. **(E)** Rise-time comparisons for A300T-containing channels. Empty circles represent mean values, boxes represent SEM values; full circles represent raw data from WT (*n* = 6), A300T (*n* = 3), P535T (*n* = 4), P535T/A300T (*n* = 8), A300T/WT (*n* = 3), and P535T/WT (*n* = 5) cells.

Two different types of use-dependent responses were observed (Figure 3A). First, the WT use-dependent response can be described as a constant current amplitude during the step and the 70 repetitions, which was also observed in P535T and P535T/WT channels. Second, an A300T use-dependent response, characterized by a current that increases in amplitude with step duration, and with each step repetition. This response was found in A300T, P535T/A300T, and A300T/WT channels.

We then plotted the end-step normalized current as a function of the step number (Figures 3B–D). To evaluate the kinetics of these responses, rise time was measured for all homo and heterotetrameric channels. WT use-dependent responses were estimated as zero because the amplitude was constant and not included in the analysis (Figure 3E). Rise time was slowest for A300T homotetrameric currents (31.89 ± 1.84 s, *n* = 3), followed by P535T/A300T (18.14 ± 3.18 s, *n* = 8) and by A300T/WT heterotetrameric currents (10.33 ± 3.86 s, *n* = 3); however, only differences between A300T homotetrameric and A300T/WT heterotetrameric channels reached statistical significance (*p* = 0.016, Figure 3E).

### The P535T Mutation Decreases Kv7.1-Plasma Membrane Colocalization

**Figure 4:**
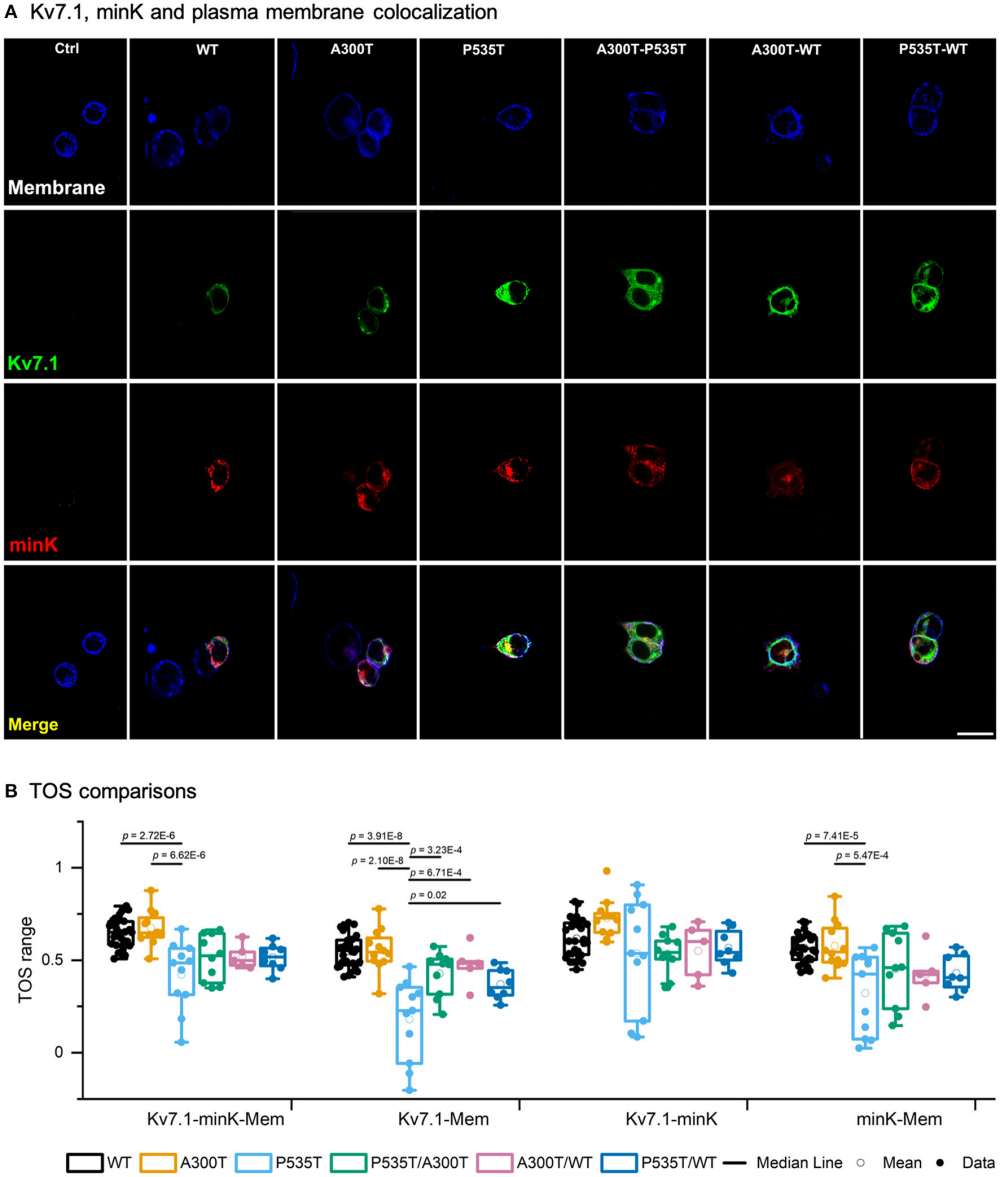
Kv7.1-plasma membrane colocalization. **(A)** Confocal images of representative HEK293 cells transiently transfected with WT-GFP, A300T-GFP, or P535T-GFP (homo and heterotetrameric channels), KCNE1-RFP and stained plasma membrane (blue signal). The final row shows all 3 signals merged. 20 μm scale applies to all panels. **(B)** Box plots of multiple threshold overlap score (TOS) comparisons of Kv7.1, minK and plasma membrane colocalizations. Full circles represent raw data, empty circles mean and boxes ± SEM; *p* values of significant differences are indicated.

Our previous Kv7.1-A300T and -P535T protein model predicted defective trafficking of the Kv7.1-P535T potassium channel (15). We used colocalization assays to further investigate if the decreased conductance density of P535T channels (Figure 1) could be due to channel density reduction at the plasma membrane (Figure 4). Table 3 describes TOS for colocalization of Kv7.1-minK-plasma membrane, Kv7.1-plasma membrane, Kv7.1-minK and mink-plasma membrane for all different homo and heterotetrameric channels. First, Kv7.1, minK, and plasma membrane (Kv7.1-minK-Mem) colocalization scores were similar for WT, A300T, and A300T/WT channels. However, all P535T-containing channels had significantly lower colocalization scores (*p* < 0.04) compared with WT, A300T, and A300T/WT channels. Second, for Kv7.1-plasma membrane (Kv7.1-Mem) colocalization, P535T channels clearly had the lowest score, being significantly lower than all other homo and heterotetrameric channels (*p* < 0.02). P535T/WT channels showed an intermediate colocalization score between WT and P535T channels, being significantly lower than the WT and A300T channels. Third, TOS comparisons of Kv7.1 and minK subunit colocalization (Kv7.1-minK) showed that all channels containing P535T monomers (P535T, P535T/WT, and P535T/A300T) were slightly lower than WT channels, however differences were not statistically significant. Finally, minK subunit-plasma membrane colocalization scores (minK-Mem) were highest for WT and A300T channels and lowest for P535T channels. Only comparisons between P535T and WT or A300T channels showed statistically significant differences (Figure 4).

**Table 3:** Threshold overlap score (TOS) (mean values ± SEM) for multiple colocalization comparisons.

	**Kv7.1-MinK-Mem**	**Kv7.1-Mem**	**Kv7.1-MinK**	**MinK-Mem**	***n***
WT	0.65 ± 0.02	0.55 ± 0.02	0.62 ± 0.02	0.57 ± 0.02	28
A300T	0.67 ± 0.03	0.56 ± 0.03	0.73 ± 0.03	0.58 ± 0.04	12
P535T	0.42 ± 0.06^†^	0.18 ± 0.07^*^	0.54 ± 0.09	0.32 ± 0.07^†^	11
P535T/A300T	0.51 ± 0.04	0.43 ± 0.04	0.54 ± 0.03	0.45 ± 0.06	10
A300T/WT	0.52 ± 0.03	0.47 ± 0.05	0.55 ± 0.07	0.42 ± 0.06	5
P535T/WT	0.52 ± 0.03	0.37 ± 0.03	0.57 ± 0.03	0.43 ± 0.04	8

* *P535T homomeric channel colocalization scores for Kv7.1-Mem were significantly lower than those of all other homo and heterotetrameric channels (p < 0.02)*.

† *P535T homo and heterotetrameric channel colocalization scores for Kv7.1-MinK-Mem and MinK-Mem were significantly lower than those of WT and A300T homotetrameric channels (p < 5.4E-4)*.

## Discussion

Next generation sequencing has facilitated the identification of genetic mutations of cardiac ion channels as a possible cause of arrhythmias. Functional information of identified genetic mutations is of aide for classification of pathogenicity, that will impact diagnosis, prognosis, and risk stratification. Moreover, molecular diagnosis may have implications in clinical decisions and is crucial for the identification of relatives at risk of sudden death (20, 21). Unfortunately, missense genetic variants are frequently classified as VUS, meaning there are insufficient data to define whether they are disease-causal or benign. We recently reported the case of a child with sudden death, who was compound heterozygous for *KCNQ1* mutations (P535T/A300T). The P535T mutation was initially classified as of unknown clinical significance, and although the electrophysiological properties of A300T channels in *Xenopus* oocytes showed clear anomalies, interpretations of these anomalies and the clinical implications of the A300T mutation have been inconsistent (16, 22–24).

Remarkably, it has been observed that mutations that are dysfunctional at the molecular level may not cause clinical disease, and alternatively, some *KCNQ1* mutations reported in LQTS patients do not show electrophysiological alterations (22). In the latter case, it is important to establish whether the variant is in fact not causal of disease, or whether the variant affects other yet unassessed biophysical properties and thus contributes to the phenotype. Most functional studies only analyze IKs amplitude and activation V_1/2_, and few include activation and deactivation time constant (τ) values. We thus further characterized the biophysical properties of A300T and P535T homo and heterotetrameric channels, analyzing prototypical activation curve parameters, current density, rise-time_300ms_, and use-dependent responses, in an effort to gain further insight into how these mutations affect the IKs and may cause LQTS.

### Kv7.1 A300T Electrophysiological Phenotype

The *KCNQ1*-A300T mutation (rs120074187) is located at the pore domain, in the P-loop between S5 and S6 transmembrane domains, a topological location with high probability of pathogenicity (25). Previously functional data in *Xenopus* oocytes indicated that the A300T Kv7.1 channel was normally transported to the cell surface but activates IKs more rapidly, left-shifts the activation voltage dependence and decreases current amplitude (16, 23), suggesting that the A300 residue plays an important role in the activation voltage dependence as predicted by structural models (26). Our colocalization data are in agreement with findings in *Xenopus* oocytes as the TOS were similar for A300T and WT channels (Figure 4). Our findings in HEK293 cells also agreed with a left-shifted activation and decreased end-step current density (Figure 1D). A300T voltage dependence was rescued when expressed together with the WT subunit (A300T/WT, Figure 1B2), compatible with a recessive biophysical trait.

We observed that tail maximum conductance density was similar in WT and A300T channels (Figure 1C2). Thus, decreased steady state current density (Iss/Cm) and normal tail maximum conductance density suggest higher deactivation extent of A300T compared with WT currents. According to our data, reduction in current density of A300T channels was not as prominent as previously reported by Priori et al. (16). This discrepancy is most likely due to differences between heterologous expression systems (*Xenopus* oocytes vs. HEK293 cells). Post-translational processing, plasma membrane composition, and multimeric protein assembly in *Xenopus* oocytes can be different from mammalian cells (27, 28). These differences should be considered for studies aimed at understanding the mechanism of native human ion channels, receptors, and their modulators.

A previous study in *Xenopus* oocytes described that A300T activation assessed over a 4–5 s period is faster (16). In the present study, we assessed WT and mutant channel IKs activation within the physiological human ventricular action potential duration range (rise-time_300ms_). This analysis revealed an A300T dominant trait described here for the first time. We observed that currents from all A300T-containing channels had significantly slower activation than WT and P535T homo and heterotetrameric channels (Figure 2). Since slower A300T activation would be expected to delay action potential repolarization, this trait can be considered as decreased function. Moreover, A300T homomeric current activation is left-shifted, meaning that it is prematurely activated during the ventricular action potential, but because this premature activation is slow, the final result could be a delayed repolarization. Additionally, on repeated activation simulating a normal heart rate, the A300T channels showed use-dependent current potentiation, which slowly reached a steady state (Figure 3). The latter characteristic was also dominant, as it was observed in currents from both A300T/WT and P535T/A300T channels (Figure 3).

Summarizing, A300T causes a recessive, increased function biophysical trait (left-shifted activation voltage), concurrently with two dominant, decreased function biophysical traits (slow 300 ms activation and use-dependent response), likely resulting in a mild overall effect. It was first described as causal of a recessive form of Romano Ward in a homozygous child with normal hearing (16). To date, all reported heterozygous A300T mutation carriers have normal QTc intervals, even after exercise (15, 16). Although the 300T allele is rare, it is most frequent in Latino populations (0.00026, https://gnomad.broadinstitute.org/).

### P535T Reduces Kv7.1 Function and Colocalization With the Plasma Membrane

The P535T mutation was originally reported by our group (15). Although it was not functionally characterized, an *in silico* model predicted that P535T disrupts a calmodulin binding site by steric hindrance, most likely causing co-assembly and/or trafficking defects. P535T is localized within the C-terminus domain (15, 29), considered with high probability for pathogenicity (25), and lies within a highly conserved protein region (22). Calmodulin is essential for correct channel folding, assembly, and trafficking, and the Kv7.1 C-terminus includes two calmodulin binding sites (30–33). Altogether, these data suggest that mutations located at this region could disrupt Kv7.1-calmodulin interactions resulting in defective trafficking. In consistency with the model predictions, we observed decreased colocalization of P535T-containing channels with the plasma membrane (Figure 4).

Although P535T-plasma membrane colocalization score was considerably lower for homotetrameric channels, colocalization analysis and electrophysiological recordings indicated the P535T mutation does not abolish Kv7.1 channel function. First, colocalization scores with plasma membrane suggested that P535T and P535T/WT channel densities were ~1/3 and 2/3 that of WT channels, respectively. Second, maximum conductance density of P535T channels was ~2/5 of that of WT channels, likely due to the lower P535T channel density in the plasma membrane. This was a recessive trait, as P535T/WT, P535T/A300T, and WT channels showed similar Gmax/Cm values (Figures 1B1,B3,C2). Similarly, other *KCNQ1* genetic mutations within this region have been associated with recessive Romano-Ward syndrome (30, 34). Finally, P535T did not alter activation voltage dependence, rise-time_300ms_ or use-dependent response. In physiological conditions, adrenergic stimulation enhances IKs and shortens ventricular repolarization, providing physiological protection against the possibility of reentrant arrhythmias at fast heart rates (35). Thus, partial IKs impairment is compatible with QT prolongation only during exercise, as observed in P535T/WT heterozygous mother (15).

### *KCNQ1* P535T/A300T Compound Heterozygosity

Because an undiagnosed child with sudden death was found to be compound heterozygous for *KCNQ1* mutations (P535T/A300T) (15), we functionally characterized the IKs of HEK293 cells expressing both mutations to establish whether she was affected with a form of recessive Romano-Ward syndrome.

#### Biophysical P535T/A300T Phenotypes

While A300T left shifted activation voltage dependence behaved as a recessive trait when co-expressed with WT alleles, it was dominant when co-expressed with P535T alleles. Although A300T/WT and WT channels showed similar activation V_1/2_ values, P535T/A300T channels showed intermediate activation V_1/2_ values, with a left-shift of 16.3 mV (*p* = 0.017) compared with P535T channels, and a right-shift of 13.8 mV compared with A300T (*p* = 0.013). Because P535T channels showed significantly lower colocalization scores with membrane than WT and A300T channels, it is likely that in a compound heterozygous state, a lower proportion of P535T and higher proportions of P535T/A300T and A300T channels will be assembled at the plasma membrane. This is compatible with the dominance of left-shifted activation voltage only in the compound heterozygous state. Finally, while P535T channels had significantly lower Gmax/Cm compared with WT channels, Gmax/Cm of P535T/A300T channels was also lower, although the difference did not reach statistical significance. These characteristics would most likely lead to abnormal repolarization and low possibilities of functional recovery since no Kv7.1-WT monomers can compensate the effects of the mutations.

### Study Limitations

Although HEK293 cells have been widely used as host for heterologous expression and functional characterization of ion channels, they do not resemble the native cardiomyocyte environment where other ion channels and intracellular molecules interact to generate action potentials. For this reason, we cannot rule out that ion channels in their native environment are subject to regulation and interactions not considered in our model. Moreover, characterization of other yet unexplored biophysical traits such as response to PKA activators simulating adrenergic stimulation will provide further insight to better understand the mechanisms by which genetic mutations cause disease, and need to be performed in future studies.

### Concluding Remarks

Co-expression of different combinations of WT, A300T, and P535T Kv7.1 with minK in mammalian cells revealed two previously undescribed biophysical phenotypes of the A300T mutation (slow rise-time_300ms_, and a characteristic use-dependent response) that behaved as dominant traits. The A300T left-shifted activation V_1/2_ previously observed in *Xenopus* oocytes behaved as a recessive trait when co-expressed with WT alleles, but as a dominant trait when co-expressed with P535T alleles. The P535T variant significantly decreased maximum conductance density and Kv7.1-minK-plasma membrane colocalization, although how the P535T mutation affects trafficking remains to be elucidated. A lower density of P535T Kv7.1 monomers at the plasma membrane is compatible with the lower current density of P535T channels and with the dominance of A300T left shift voltage activation observed only when combined with P535T. The biophysical properties of P535T/A300T IKs are compatible with recessive Romano-Ward syndrome. Further analysis of other biophysical traits may identify other mechanisms involved in the pathophysiology of this disease.

## Data Availability Statement

The datasets presented in this study can be found in online repositories. The names of the repository/repositories and accession number(s) can be found at: https://www.ncbi.nlm.nih.gov/snp/, rs120074187; https://www.ncbi.nlm.nih.gov/clinvar/variation/3128/, rs120074187; https://gnomad.broadinstitute.org/, rs120074187; https://varsome.com, rs120074187; https://varsome.com, chr11-2797202-C-A (KCNQ1:p.P535T).

## Author Contributions

TV-M and AC conceptualized and designed the study. TV-M and AG-G designed the experimental approach and drafted the manuscript. AG-G performed all experiments and analyzed the data. MD-P performed confocal experiments. OL-R contributed to the data analysis and comments to the manuscript. LJ-A, JG-C, and OZ-G performed the experiments. MD-P, LJ-A, PI, and AC contributed to the manuscript. All authors contributed to the article and approved the submitted version.

## Conflict of Interest

The authors declare that the research was conducted in the absence of any commercial or financial relationships that could be construed as a potential conflict of interest.
